# Editorial: Overlap of Neural Systems for Processing Language and Music

**DOI:** 10.3389/fpsyg.2016.00876

**Published:** 2016-06-14

**Authors:** McNeel G. Jantzen, Edward W. Large, Cyrille Magne

**Affiliations:** ^1^Language and Neural Systems Laboratory, Department of Psychology and Behavioral Neuroscience Program, Western Washington UniversityBellingham, WA, USA; ^2^Music Dynamics Laboratory, Department of Psychology, University of ConnecticutStorrs, CT, USA; ^3^Brain and Language Laboratory, Department of Psychology and Interdisciplinary Program in Literacy Studies, Middle Tennessee State UniversityMurfreesboro, TN, USA

**Keywords:** musical training, language, speech, music, auditory perception, neural plasticity, development, neural overlap

The relationship between musical training and speech perception has intrigued researchers in language and music for decades, from Bever and Chiarello's (1974) work emphasizing hemispheric specialization to Tallal and Gaab's (2006) findings of shared neural circuitry. Recent studies demonstrating neural overlap for processing speech and music, and enhanced speech perception and production in musicians, suggest that these regions may be inextricably intertwined (Sammler et al., 2007; Wong P.C. et al., 2007; Wong P. et al., 2007; Rogalsky et al., 2011; Schulze et al., 2011). Patel's OPERA hypothesis and Hickok and Poeppel's (2000, 2007) neuroanatomical models continue to evolve and guide this field of research. However, the extent of neural overlap between music and speech remains hotly debated (Norman-Haignere et al., 2015; Peretz et al., 2015), with surprisingly little empirical research exploring specific neural homologs and analogs. Emerging evidence suggests that shared processes likely exist throughout development, depend upon an individual's acoustic experiences, and are affected by developmental trajectories. Moreover, developing theories that address the neural and developmental interaction between music and language processing in conjunction with the broad availability of sophisticated tools for quantifying brain activity and dynamics offer the perfect opportunity for researchers to address these key empirical questions. Taken together, this field of research has begun to elucidate the complex dynamics of overlapping neural areas for processing language and music. This special issue highlights the development of this overlap in early childhood and explores how the interaction between language and musical training enhances cognitive functioning in adults.

This E-Book comprises 10 opinion, perspective, and research papers that focus on the overlap of neural systems for processing language and music. Eight of these papers report original research and new findings that support overlapping neural systems for processing language and music. LaCroix et al. performed a meta-analysis of 171 neuroimaging studies to examine the role of context in processing music and language. Their findings suggest that observed neural overlaps for speech and music might be task-dependent. Fogel et al. developed a novel method for studying and quantifying predictions in musical tasks that is consistent with language tasks. Their melodic cloze probability task can be used to test computational models of melodic expectation and allows for a more precise examination of the relationship between predictive mechanisms in music and language. Using a garden-path design, Jung et al. demonstrated that rhythmic expectancy is crucial to the interaction of processing musical and linguistic syntax. Additionally, their findings support the incorporation of dynamic models of attentional entrainment into existing theories of musical and linguistic syntactical processing. Margulis et al. used the speech-to-song illusion to examine the role of pronunciation difficulty and temporal regularity. Their finding—that difficult to pronounce languages, not differing temporal intervals, elicited a stronger speech-to-song illusion—suggests a stronger speech representation for native and easy to pronounce languages. Miles et al. demonstrated that females have an advantage for recognizing familiar musical melodies. They believe this advantage is related to superior declarative memory, which may underlie the storage and knowledge of both the mental lexicon in language (e.g., Ullman, 2001) and some aspects of familiar melodies in music (Miranda and Ullman, 2007). Two papers report finding that musical training during development enhances literacy skills, including phonological awareness and reading fluency, via neural mechanisms for both language and music (Degé et al.; Gordon et al.). Moreover, Degé and colleagues provide evidence that music production and music perception are associated with multiple precursors of reading. Finally, Lolli et al. examined the effect of sound frequency on judgments of emotion in speech by congenital amusics. Using both high and low-pass filtered speech in a pitch discrimination and emotion identification task, their findings demonstrate the important role of low frequency information in identifying the emotional content of speech.

In addition to these eight research papers there are two perspective and opinion papers that emphasize the affective and emotive commonalities between music and language (Lehmann and Paquette; Omigie). Lehmann and Paquette provide a neurobehavioral approach for examining cross-domain processing of musical and vocal emotions, suggesting that studying cochlear implant users may allow for a richer understanding of neural overlap between music and language. Omigie (2015) provides evolutionary evidence for shared underlying neural mechanisms for our emotive responses to music and literature.

This E-Book provides a comprehensive snapshot of the research examining the complex overlap of neural systems for processing language and music. Both musical experience and training enhance the development of linguistic representations, emotion perception, and other cognitive skills. Furthermore, the research presented here contributes to current knowledge of neuroplastic reorganization and repair in clinical populations, and may aid in the design of new and more effective rehabilitative protocols.

## Author contributions

MJ prepared and wrote the editorial soliciting feedback from EL and CM. EL and CM provided feedback regarding organization as well.

### Conflict of interest statement

The authors declare that the research was conducted in the absence of any commercial or financial relationships that could be construed as a potential conflict of interest.
